# Cerebral and Nerve Surgery

**Published:** 1904-12-03

**Authors:** 


### CEREBRAL AND NERVE SURGERY.

**The Surgical Treatment of Epilepsy.**—Da Costa[1] states that less than 5 per cent. of epileptics are permanently benefited by an operation. More than three years should elapse without recurrence of fits before the case is pronounced cured. Uncomplicated cases of essential epilepsy should not be operated upon. Cases of epilepsy with focal symptoms are only amenable for operation if taken within two years from their onset, and if the symptoms have been focal from the first. Epilepsy in a generalised form only follows traumatism when there is a strong neurotic heredity. Jacksonian epilepsy gives a better prognosis from operation than any other form.

**On the Path and Nature of Cerebral Infection.**—Macewen[2] refers to the path by which infection of cerebral structures takes place, and points out that the source and also nature of the infection bears a constant relation to the resulting infection. Thus, while septic invasion of the internal auditory meatus results in a basal meningitis, that which occurs through the sigmoid or tegmen produces a localised meningitis or a cerebral or cerebellar abscess. Ten cases of tubercular meningitis following tuberculosis of the middle ear are mentioned, in five of which intracranial operations were performed in the hope that the meningitis was still limited in area. In three of the five this hope was rewarded by success.

**Transplantation of Tendon for Musculo-Spiral Paralysis.**—Gray[3] describes a case of a boy of 16, who after compound fracture of the humerus had complete musculo-spiral paralysis, and in whom direct operation for freeing the nerve had failed. He transplanted the tendon of the flexor ulnaris through an opening in the interosseous membrane and fixed it to the tendons of the common extensor. Immediate and complete success in enabling the boy to extend his fingers was the result.

**The Treatment of Tetanus by Antitoxin.**—Charpentier[4] describes a case of tetanus in which 2 cubic centimetres of antitoxin were injected into each side of the brain, but in which the patient died in spite of the fact that all tetanic spasms ceased after the injection. He attributes death to septicæmia, the original injection being "mixed." Köhler is quoted as stating that while 86 per cent. of cases of tetanus untreated by antitoxin die, that only 30 per cent. of those treated by intracerebral injection of the antitoxin succumb. John Rogers[5] refers to work showing that tetanus exerts its effects entirely on the cells of the central nervous system, and chiefly on those of the cord, and further that the toxin is absorbed by the muscle plates of the motor nerves and finds its way to the central nervous system only by traversing axis cylinders in a centripetal direction. The antitoxin takes the same course. Neither toxin or antitoxin take a centrifugal, but only a centripetal direction, and in the central nervous system itself flow from the lowest end of the cord upwards to the brain. From these considerations the deduction is made that for the antitoxin to take the most rapid effect it should be injected (1) into the nerves of the injured part, and (2) into the spinal cord by the usual lumbar puncture. One case in which this was successfully carried out is described.

**Recovery from Septic Meningitis and Hernia Cerebri.**—Mr. Bucknall[6] describes the case of a boy 16 years old who had a compound comminuted fracture of the skull in the left parietal region. Operation 30 hours after. Many fragments of bone were removed; the dura was found punctured with bone driven through it. A large piece of bone was removed in order to tie the meningeal artery. During the following week, high temperature, great restlessness and delirium, frequent fits and vomiting, and severe headache occurred, with transient paralysis and optic neuritis. A hernia cerebri rapidly formed and protruded from the wound from which much pus exuded. After a fortnight he began to recover rapidly and was quite well two months after the accident, the hernia having shrunk. The recovery from the septic meningitis, and the subsidence of the hernia cerebri, is referred to the large opening made in the skull which gave free drainage and prevented too great rise of intracranial pressure.

**Operative Treatment of Cranial Depression.**—Henderson Nicholl[7] describes six consecutive successful cases of operation for fracture of the skull in infancy. Four were birth fractures and two resulted from injury during infancy. The method adopted in all was the same. The depressed area of bone was cut out by a large trephine one-and-a-half inches to two-and-a-half inches diameter after reflection of the skin and pericranium. The separated circle of bone was then inverted and the skin sewn over. The bone united very rapidly in all cases.

[1] Medicine, February, p 96. [2] Brit. Med. Jour. July 30. [3] Lancet, May 21. [4] Lancet, June 18. [5] Med. Rec., May 21. [6] Lancet, July 30. [7] Brit. Med. Jour., Dec. 19, 1903.

